# Knowledge and Awareness of Parents and the General Population Living in Riyadh About Amblyopia

**DOI:** 10.7759/cureus.65451

**Published:** 2024-07-26

**Authors:** Faisal A Aldihan, Nawaf M Alamer, Abdullah Alhejji, Fahad A Al Dihan, Faisal A Alshahrani, Nawaf K Alawad, Taghreed Alnahedh, Mohammed Taha

**Affiliations:** 1 College of Medicine, King Saud Bin Abdulaziz University for Health Sciences, Riyadh, SAU; 2 Optometry, King Saud Bin Abdulaziz University for Health Sciences, Riyadh, SAU; 3 Ophthalmology, King Saud Bin Abdulaziz University for Health Sciences, Riyadh, SAU

**Keywords:** amblyopia treatment, amblyopia, refractive amblyopia, knowledge and attitudes, awareness

## Abstract

Introduction

Amblyopia is a neurodevelopmental disorder of the visual cortex that usually occurs due to abnormal visual development early in life. The clinical importance of amblyopia is that it is a significant cause of visual loss in the pediatric population. Knowing and understanding amblyopia plays a crucial role in management since the earlier the intervention, the better the results.

Aim

This study aimed to assess the knowledge and awareness of amblyopia among the general population in Saudi Arabia.

Subject and methods

This cross-sectional study was conducted among the general population in Riyadh, Saudi Arabia. A self-administered Arabic questionnaire was distributed to the general population through social media using Google Forms. The questionnaire includes sociodemographic characteristics (e.g., age, gender, marital status, etc.) and questions to assess the knowledge about amblyopia, including its definition, etiologies, and treatment.

Results

Of the 488 participants, 57.4% were females, and 38.3% were aged between 26 and 39 years old. Of the participants, 46.5% have heard of amblyopia. The overall mean knowledge score was 16.9 (SD 3.09) out of 32 points. In terms of knowledge, 64.5% had moderate knowledge, 1.8% had good knowledge, and 33.6% had poor knowledge levels. Factors associated with increased knowledge were being older, female gender, married, having children, having a family history of eye disease, and hearing of amblyopia.

Conclusion

Consistent with the literature, this study finds a need for more knowledge about amblyopia among the general population. Significant predictors of increased knowledge include younger age group, female gender, having children, family history of eye disease, and ever heard of amblyopia.

## Introduction

Amblyopia is a neurodevelopmental disorder of the lateral geniculate body and visual cortex that usually occurs due to abnormal visual development within the first three to eight years of life. It is a major cause of visual loss in the pediatric population, which is the reason for the clinical importance of this condition. Additionally, amblyopia reflects the neural impairment that occurs when normal visual development is impaired, making the topic a base of interest in ophthalmology [1]. In developing countries, amblyopia is the second most common cause of visual impairment in children. In the United Kingdom, it is the most common cause of vision impairment in the pediatric population [2]. According to a study conducted in Saudi Arabia, with a sample of 15,281 children pooled from seven studies, the estimated prevalence of amblyopia was 2.3% [3].

To have normal vision development, it is important to have binocular single vision (BSV), in which both eyes must receive equally clear, focused images simultaneously to achieve a single-image perception. Lack of BSV early in life could lead to the suppression of the amblyopic eye by the other eye. Insufficient visual stimulation during the crucial period of visual development can lead to amblyopia [4]. In patients with amblyopia, many manifestations could be found in history and examination, including reduced visual acuity during Snellen, grating, and vernier tests. Also, there is an absence of sensitivity to the contrast in a stimulus, distortions in the shape of a stimulus, a lack of sense of the position of a stimulus, mobility deficits, and a rise of the crowding effect or separation difficulty [5]. In terms of visual acuity, there are three periods: the period when a child develops their visual acuity from less than 20/200 to nearly 20/20 (birth to three or five years of age), the period when deprivation causes amblyopia (from a few months to seven or eight years of age), and the critical period during which amblyopia can be recovered (adolescent years up to adulthood) [5]. Furthermore, visual cortex plasticity peaks during the critical period, which signifies the early intervention to correct the neurodevelopmental deficit [1]. Despite that, animal testing and clinical trials have shown potential for recovery even after the critical period of brain plasticity [6-8].

Amblyopia can be categorized by the causes that lead to the loss of BSV. Strabismus amblyopia occurs when there is a misalignment of the eyes, in which the brain will suppress one eye to avoid diplopia, leading to impaired visual development in that eye. Refractive amblyopia occurs when there is a significant difference in the refractive error between the two eyes that is usually greater than 1 diopter. Lastly, deprivation amblyopia is caused by any partial or complete obstruction of the visual pathway, leading to impaired BSV. This can be due to corneal opacity, congenital cataracts, inflammation, or retinal pathology [4].

Amblyopia should be managed as early as possible, as one clinical trial found a better outcome for children if treatment started at a young age [9]. Causes of visual deprivation, such as cataracts, must be treated first. Moreover, cases of refractive amblyopia should be thoroughly assessed and prescribed appropriate lenses to correct their refractive error [4]. If amblyopia develops, patching the good eye has good outcomes with compliance and initiation at an early age. In 246 amblyopia patients treated with eye patches, therapy succeeded for 85% of those with strabismic amblyopia and 100 percent in those with anisometropia [10]. Moreover, atropine has a role in the management of amblyopia. It can be used in the non-affected eye, which causes pupillary dilation, leading to ciliary body paralysis, and eventually blurry vision in that eye. Therefore, the amblyopic eye gains an advantage and is encouraged to be used [11].

Knowing and understanding amblyopia plays a key role in management since the earlier the treatment is administered, the better the results. Locally, in one study, only 20% of participants in Jeddah city reported to have sufficient knowledge of amblyopia [12]. A second local study in Hail City showed a need for more awareness and knowledge [13]. Among 1,649 participants in a recent large study in Saudi Arabia, only 495 (30%) parents were aware of amblyopia [14]. Therefore, the study aimed to evaluate the knowledge and awareness of amblyopia among the general population of Riyadh city and to compare the knowledge of parents with the general population.

## Materials and methods

This cross-sectional study was conducted in Riyadh, Saudi Arabia, to assess the knowledge and awareness about amblyopia of the general population living in Riyadh. The study targeted those aged 18 years and above. Moreover, those who were not a resident of Riyadh were excluded. The questionnaire was distributed using Google Forms through social media (convenience sampling), in which the data were exported to Microsoft Excel. Data that presented categorical variables were expressed as percentages and frequencies, whereas continuous variables were presented as means and standard deviations. Moreover, according to the General Authority for Statistics, population of Riyadh is almost 8.6 million. The required sample size was calculated using Raosoft (Raosoft Inc., Seattle, WA). It was calculated to be 385 with a confidence level of 95% and a margin of error of 5%. The ethical approval was granted by the Institutional Review Board of King Abdullah International Medical Research Center, Riyadh, Saudi Arabia.

Questionnaire structure

The Arabic questionnaire that was used in this research was adopted from Almutairi et al. [13]. Initially, a statement describing the research's purpose and goals was provided to the participants in which they would express their consent to participate with a “Yes” or “No.” The first section of the questionnaire gathered demographic information, including age, gender, city of residence, and educational level. In the second part of the questionnaire, participants were asked multiple-choice questions to assess their knowledge about amblyopia, including its definition, etiology, and management.

Questionnaire criteria

Knowledge about amblyopia was assessed using a 32-item questionnaire. The correct answer for each item was identified and coded with 1, while the incorrect answer was coded with 0. The total knowledge score was calculated by adding all 32 items. A possible score ranging from 0 to 32 points was generated. The higher the score, the higher the knowledge of amblyopia. The cutoff points were 50% and 75% to determine the knowledge levels. Respondents were considered as having poor knowledge if the total score was <50%, as having moderate knowledge if the score was 50% to 75%, and as having good knowledge level if the score was above 75%.

Statistical analysis

Categorical variables were described using counts and proportions (%), while continuous variables were computed and expressed using means and standard deviations. The differences in the knowledge score in relation to participants' sociodemographic characteristics were assessed using the Mann-Whitney Z-test. The normality test was calculated using the Shapiro-Wilk test and the Kolmogorov-Smirnov test. The knowledge score followed the non-normal distribution. Thus, the non-parametric tests were applied. A p-value of less than 0.05 was considered statistically significant. All statistical data were analyzed using Statistical Packages for Social Sciences (SPSS) Version 26 (IBM Corp., Armonk, NY, USA).

## Results

A total of 488 participants completed the survey (Table 1). More than one-third of the participants (38.3%) were between 26 and 39 years of age, with more than half (57.4%) being females. In terms of marital status, 48.6% were married and 47.5% had children. In terms of education, 58.8% had a bachelor's degree. The prevalence of participants with a family history of eye disease was 41.2%. In addition, 46.5% reported having heard of amblyopia.

**Table 1:** Sociodemographic characteristics of participants (n=488) N, number of participants

Study variables	N (%)
Age group	
18–25 years	151 (30.9%)
26–39 years	187 (38.3%)
40–60 years	133 (27.3%)
>60 years	17 (03.5%)
Gender	
Male	208 (42.6%)
Female	280 (57.4%)
Marital status	
Single	229 (46.9%)
Married	237 (48.6%)
Divorced	16 (03.3%)
Widowed	06 (01.2%)
Having children	
Yes	232 (47.5%)
No	256 (52.5%)
Educational level	
Primary school	12 (02.5%)
Secondary school	88 (18.0%)
Diploma holder	33 (06.8%)
Bachelor's degree	287 (58.8%)
Postgraduate	68 (13.9%)
Family history of eye disease	
Yes	201 (41.2%)
No	238 (48.8%)
I don't know	49 (10.0%)
Heard of amblyopia	
Yes	227 (46.5%)
No	247 (50.6%)
I don't know	14 (02.9%)

Regarding the assessment of the knowledge about amblyopia (Table 2), it was revealed that only 17.8% were correct that the naked eye cannot detect amblyopia. Also, only 29.3% knew that the general pediatric or family doctor could not diagnose amblyopia, but 53.9% knew that eye specialists could diagnose amblyopia instead. Only 15.6% knew that children are more prone to amblyopia. Regarding the knowledge of amblyopia definition, the majority disagreed that squint (85%), decreased night vision (78.3%), the inability of the eye to move (75%), and abnormal eye movement (69.9%) were the correct definitions of amblyopia, while only 27% were aware that eye and brain not working well together and only 10.7% agreed that vision loss in one eye were the correct definitions of amblyopia. However, 87.5% were aware that it is important to check a child's vision before school for normal development. In regard to the best period to treat amblyopia, 42.7% of the participants didn't know the answer and 21.5% answered that either there is no specific period to treat it or that it could be treated after the critical period, while only 28.3% were correct that the best age period for the treatment of amblyopia was between three and nine years. Most participants were unaware of the etiologies of amblyopia, as their understanding of the correct etiologies was poor, particularly refractive error (31.4%), cataract (20.7%), prematurity (16.2%), and Down syndrome (7%.) Regarding the knowledge about amblyopia treatment, most of the participants (95.1%) knew that amblyopia cannot resolve spontaneously and that laser therapy is not a treatment method for amblyopia (71.1%). However, only 36.1% and 35.5% answered correctly that glasses and patches on the healthy eye are the treatment methods for amblyopia. Based on the above items, the total mean knowledge score was 16.9 (SD 3.09), with poor, moderate, and good knowledge levels constituting 33.6%, 64.5%, and 1.8%, respectively.

**Table 2:** Assessment of the knowledge about amblyopia (n=488) N, number of participants

Variables	N (%)
General information about amblyopia	
Amblyopia can be detected by the naked eye (no)	87 (17.8%)
Amblyopia can be diagnosed by the general pediatric or family doctor (no)	143 (29.3%)
Amblyopia can only be diagnosed by an eye specialist (yes)	263 (53.9%)
Who are exposed to amblyopia? (children)	76 (15.6%)
Knowledge about amblyopia definition	
Squint (no)	415 (85.0%)
Decreased night vision (no)	382 (78.3%)
Inability of the eyes to move (no)	366 (75.0%)
Abnormal eye movement (no)	341 (69.9%)
Decreased vision in one or both eyes (yes)	238 (48.8%)
Degeneration of optic nerve (no)	230 (47.1%)
Eye and brain not working well together (yes)	132 (27.0%)
Vision loss in one eye (yes)	52 (10.7%)
Important to check a child's vision before school for normal development (yes)	427 (87.5%)
What is the best age period for the treatment of amblyopia?	
After the age of 10 years	11 (2.2%)
before the age of 1 year	37 (7.4%)
Between 3 to 9 years	138 (28.3%)
There is no specific age period	97 (19.3%)
I don't know	215 (42.7%)
Knowledge about amblyopia etiologies	
Cerebral palsy (no)	432 (88.5%)
Sunlight exposure (no)	428 (87.7%)
Maternal illness (no)	411 (84.2%)
Fever in infancy (no)	409 (83.8%)
Nutrition deficiency (no)	409 (83.8%)
Trauma (no)	344 (70.5%)
Electronic devices use (no)	331 (67.8%)
Hereditary cause (no)	165 (33.8%)
Refractive error (yes)	153 (31.4%)
Cataract (yes)	101 (20.7%)
Prematurity (yes)	79 (16.2%)
Down syndrome (yes)	34 (07.0%)
Knowledge about amblyopia treatment	
Resolve spontaneously (no)	464 (95.1%)
Laser therapy (no)	347 (71.1%)
Surgical intervention (no)	283 (58.0%)
Eye muscle exercise (no)	206 (42.2%)
Glasses (yes)	176 (36.1%)
Patch on the healthy eye (yes)	173 (35.5%)
Total knowledge score (mean ± SD)	16.9 ± 3.09
Level of knowledge	
Poor	164 (33.6%)
Moderate	315 (64.5%)
Good	09 (01.8%)

**Figure 1:**
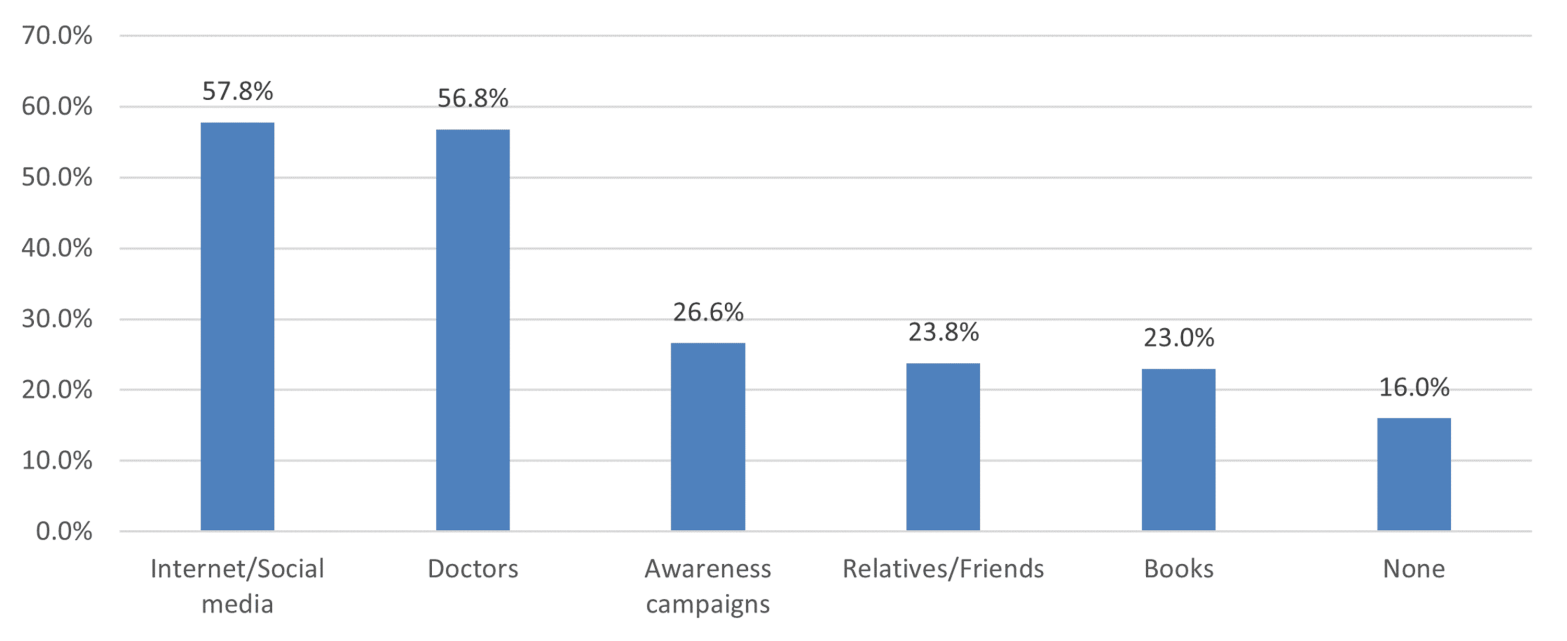
Sources of amblyopia information

According to multiple response answers, the most common source of amblyopia information was the Internet/social media (57.8%), followed by doctors (56.8%) and awareness campaigns (26.6%) (Figure 1).

When measuring the differences between the score of knowledge and the sociodemographic characteristics of participants (Table 3), it was observed that a higher knowledge score was more associated with being older (Z=4.594; p<0.001), female gender (Z=2.906; p=0.004), being married (Z=3.912; p<0.001), having children (Z=4.035; p<0.001), having a family history of eye disease (Z=2.095; p=0.036), and ever heard of amblyopia (Z=6.725; p<0.001), while a lower knowledge score was more associated with having no source of amblyopia information (Z=2.075; p=0.038). Also, the sources of amblyopia information such as awareness campaign (Z=2.625; p=0.009) and relatives/friends (Z=2.387; p=0.017) had lower knowledge scores.

**Table 3:** Differences in the score of knowledge according to the sociodemographic characteristics of participants ^§^P-value has been calculated using the Mann-Whitney Z-test. **Significant at p<0.05 level. SD, standard deviation

Factor	Knowledge score (32), mean ± SD	Z-test	P-value^§^
Age group in years			
<40 years	16.5 ± 3.01	4.594	<0.001**
≥40 years	17.8 ± 3.07		
Gender			
Male	16.4 ± 3.12	2.906	0.004**
Female	17.2 ± 3.02		
Marital status			
Unmarried	16.4 ± 3.09	3.912	<0.001**
Married	17.4 ± 2.99		
Having children			
Yes	17.5 ± 2.97	4.035	<0.001**
No	16.3 ± 3.10		
Educational level			
Diploma or below	16.7 ± 3.04	0.834	0.404
Bachelor or higher	16.9 ± 3.10		
Family history of eye disease			
Yes	17.3 ± 3.03	2.095	0.036**
No	16.7 ± 2.95		
Ever heard of amblyopia			
Yes	17.9 ± 3.19	6.725	<0.001**
No	15.9 ± 2.66		

## Discussion

Children with amblyopia require timely and effective intervention since it has various implications for different aspects of life including health-related aspects. The present study was carried out to evaluate the knowledge and awareness of the general population regarding amblyopia, as parental understanding of said condition is an essential component of the management plan.

Level of knowledge

Throughout the 32 questionnaire items assessing knowledge, 33.6% of participants were considered to have poor knowledge levels, similar to several published studies documenting a general lack of awareness toward amblyopia [12-20]. In India [21], however, although limited knowledge was seen among parents, their attitudes and practice patterns with amblyopia were sufficient. Also, another study in Pakistan found better awareness levels of the parents regarding child ocular diseases and care as almost 41% were well informed regarding the condition [22]. Since the results among the studies are varied, extensive awareness campaigns are necessary to bridge the knowledge gaps.

Significant factor of knowledge

Data from this study suggest that younger participants, females, married individuals, and those with a prior family history of eye disease generally had a better understanding of the condition's nature, comparable to the findings of Almutairi et al. [13]. In a study by Alsaqr and Masmali [14], knowledge level was significantly associated with parents' gender, marital status, occupation, residence region, and having children. In another study by Makhdoum et al. [19], fathers with better education, Saudi mothers, those with self-perceived knowledge of amblyopia, and parents with children previously diagnosed with amblyopia were more knowledgeable of the disease than their counterparts.

Knowledge about the basic facts of amblyopia

Our results showed that even though most of our respondents knew the importance of a child vision check-up before going to school to warrant normal development (87.5%) and more than half of the participants (53.9%) were aware that eye specialists could accurately diagnose amblyopia, their comprehension of the basic facts of amblyopia was inadequate. In more detail, shortfalls are highlighted on whether the naked eye can detect it (17.8%), whether children are susceptible to amblyopia (15.6%), and whether the general pediatrician and family doctor can diagnose it (29.3%). These also mirrored the findings of Almutairi et al. [13]. In Jazan [17], the importance of examining the child's visual acuity before school entrance to ensure normal vision development was also chosen by most study subjects. In contrast, the items regarding the causes and definition of amblyopia and the recommended number of vision screenings for a child aged 6 to 12 were chosen the least.

Knowledge about the definition of amblyopia

Many of our respondents knew that squint (85%), decreased night vision (78.3%), the inability of the eye to move (75%), and abnormal eye movement (69.9%) were not correctly reflecting an accurate definition of amblyopia. In a study that was conducted in the Aseer region [15], only 52% of parents knew that amblyopia underlines a decreased vision in one eye because the brain ignores unclear images transmitted by the affected eye, favoring the healthy one. Only 16% believe that squint is a cause of amblyopia, while 12% considered genetics. In Jeddah [18], 41.0% and 33.9% correctly recognized amblyopia as a vision loss in one eye or decreased vision in one or both eyes, respectively, with half of the respondents agreeing that amblyopia may instigate severe complications.

Knowledge about the etiologies of amblyopia

Even though most of our respondents identified false etiologies of amblyopia, such as cerebral palsy (88.5%), sunlight exposure (87.7%), and maternal illness (84.2%), their knowledge about the correct etiologies was suboptimal with refractive error (31.4%), cataract (20.7%), prematurity (16.2%), and Down syndrome (7%). In Madinah [19], parents recognized myopia and farsightedness as the most frequent causes of amblyopia, while in Riyadh [20], both refractive errors (28.6%) and cataracts (10%) were identified as the most common etiologies of amblyopia.

Knowledge about the treatment method for amblyopia

Only 36.1% and 35.5% recognized that glasses and putting a patch on the affected eye, respectively, were management options, which is unfortunate, as having a better understanding of the available options is an integral part of management. Nevertheless, 95.1% do not believe that the disease can be resolved spontaneously, and our respondents did not recognize laser therapy as a treatment method for amblyopia (71.1%). Among parents in the Aseer region [15], laser therapy was chosen by 19% of the parents; 17% chose to cover the healthy eye, and 10% believed in surgical intervention. However, among parents living in Al Ahsa [16], 26.4% perceived glasses as the best treatment method for amblyopia, followed by patching by the strong eye (13.7%).

Sources of amblyopia information

In this study, the most prominent sources of information were the Internet/social media (57.8%) and doctors (56.8%). Other less used sources were awareness campaigns (26.6%), relatives/friends (23.8%), and books (23%). These findings are consistent with the literature, which states that the Internet and eye care professionals are the leading sources of information regarding amblyopia [12-15]. In Madinah [19], almost three-quarters of the participants who obtained information from books/newspapers/magazines demonstrated adequate knowledge compared with participants who received information from medical staff (p<0.001).

Our study faces several limitations within its methodology. Using a self-administered questionnaire distributed through social media can predispose to selection bias, which can skew the results toward specific demographics, as the younger population is more likely to use social media. Moreover, convenience sampling may not be representative of the population of Riyadh as it is not randomly selected. Lastly, the wording and framing of the questionnaire could guide the participants into choosing specific answers. Further studies should address these limitations to better and more accurately understand the awareness levels in the area's population.

## Conclusions

This study investigates awareness and knowledge of amblyopia. The main finding of the study showed moderate overall knowledge of amblyopia in most of the participants. However, the majority had poor knowledge of specific aspects of amblyopia including its etiology and the appropriate intervention timing. Regarding the predictors of knowledge, younger population, females, those who are married, and parents were more likely to have better scores. Due to the need for appropriate intervention, health professionals, education centers, the media, and social organizations should expand their efforts to raise awareness of amblyopia to detect it in its early stages.
